# Takotsubo Syndrome or Peripartum Cardiomyopathy? Depends on Who You Are Talking to

**DOI:** 10.3390/bs14090777

**Published:** 2024-09-05

**Authors:** Abigail O. Falola, Naveed Razvi, Ruta Gada, David R. Thompson, Colin R. Martin

**Affiliations:** 1 East Suffolk and North Essex NHS Foundation Trust, Ipswich IP4 5PD, UK; 2 Institute of Health and Wellbeing, University of Suffolk, Suffolk IP4 1QJ, UK; 3 School of Nursing and Midwifery, Queen’s University Belfast, Belfast BT9 7BL, UK

**Keywords:** takotsubo syndrome, peripartum syndrome, apical ballooning syndrome, broken-heart syndrome, stress syndrome, clinical decision-making, structural equation modelling

## Abstract

Takotsubo syndrome (otherwise known as broken-heart syndrome or left ventricular apical ballooning) is a rare cause of reversible heart failure that predominantly affects postmenopausal women. It was first described by Japanese researchers in the 1990s and has become established as a differential for heart failure following a physically or psychologically stressful event. This was popularised by a spike in cases following natural disasters in Japan. As the recognition of takotsubo syndrome in the differential diagnosis for sudden, onset heart failure in a previously healthy individual has grown, so has the discussion concerning takotsubo in the peripartum period. Peripartum cardiomyopathy is a rare cause of reversible heart failure in the latter weeks of pregnancy and the postpartum period. Morbidity and mortality for both cardiomyopathies can be highly variable, ranging from complete recovery of cardiac function to life threatening arrhythmias and even death. This rapid review highlights the similarities between both cardiomyopathies and challenges the hitherto assumption that both takotsubo and peripartum cardiomyopathies are distinct entities that can easily be distinguished from one another. The implications of this are significant within the context of the behavioural aspects of diagnosis, treatment, and outcome.

## 1. Introduction

There has been an increase in the number of women with medical problems prior to pregnancy. This can be attributed to a variety of factors including congenital heart disease patients reaching adulthood, increasing accessibility of antenatal care, the advancement and accessibility of ART (assisted reproductive technology) for mothers over advancing years, the rising recognition of medical disorders developed during pregnancy, and the rising obesity in pregnancy, to name a few [1]. It has long been demonstrated that specialising amongst doctors leads to better care, on the assumption that greater experience leads to prompt recognition and a reduction in morbidity and mortality from health conditions. The difficulty is obstetricians and midwives who are primary care providers for pregnant women are experienced in pregnancy care but not in managing complex medical conditions as it does not form the bulk of their experiences. The landscape of obstetric care has changed over the past century through the pursuit of health equality and the national drives to reduce maternal mortality. Confidential enquires and reports such as “Mothers and Babies: Reducing Risk through Audits and Confidential Enquiries across the UK” (MMBRACE-UK) highlight suboptimal care or failure to recognise the development of medical conditions not commonly found in pregnant women has led to the untimely deaths of many women [2,3]. There are many proposed solutions to this gap in experience, one being the development of the subspecialty of maternal medicine in which the obstetrician with increased experience in medical conditions in pregnancy leads a multidisciplinary team to manage conditions in pregnancy. The aim, being high-risk women, are identified and medical specialties are invited to offer collaborative care. Obstetric medicine is one of the unique areas of medicine in which cross disciplines are highly collaborative in identifying and managing pre-existing issues within the peripartum period. However, pregnancy physiology is unique and complex, and collaborative working is difficult to achieve in every hospital. To fill that gap, a new subspecialty of medicine has arisen called “obstetric physicians”. The obstetric physician is trained in general internal medicine and subspecialises in maternal medicine [4]. NHS England published its plan for maternal medicine network services in which it highlighted the aim to concentrate highly experienced teams to specific areas to manage complex medical conditions in pregnancy and in the postpartum [5]. Many are the mitigations that the medical community have established to manage the known obstacle presented by the bias and blind spots of a care provider when diagnosing problems in pregnancy, and efforts to reduce this obstacle has been met with reasonable success [2]. When confronted with a condition that is relatively rare and can present in an obscure manner, perhaps the obstetrician will have a bias toward a specific diagnosis they are more familiar with, and the same will occur with the internal physician. One of the components of evidence-based medicine is the elimination or recognition of biases wherever possible for all avenues to be explored and proven facts to be determined. We posit that the bias that colours the researchers or clinicians view and precipitates better care can also leave us with blind spots, especially in the process of navigating opaque syndromes. 

One such opaque and frequently fatal condition is heart failure in the pregnant or peripartum period. Over the last century, although it has gained increasing awareness and publicity, it is an example of a condition that falls into the space of requiring the expertise of both the obstetrician and the physician. This is the context in which we aim to discuss peripartum and takotsubo syndrome.

### 1.1. Peripartum Cardiomyopathy

Cardiac failure, specifically left ventricular pump failure as a cause of maternal mortality and morbidity in peripartum period, has been described since the 19th century. The first landmark study and characterisation of peripartum cardiomyopathy was published by Demakis et al. in 1971. Peripartum cardiomyopathy (PPCM) was first described as a clinical syndrome of cardiomegaly and heart failure. At that time, the diagnostic criteria comprised of the development of cardiac failure in the last month of pregnancy or five months postpartum, absence of any other determinable aetiology, and absence of symptoms of demonstrable cardiac conditions prior to the last month of pregnancy [6].

Since then, several multi-centre studies and trials have worked to demystify and characterise PPCM. A landmark position statement by the Heart Failure Association of the European Society of Cardiology (ESC) working group on PPCM in 2010 appears to have one of the most widely accepted definitions of PPCM: “an idiopathic cardiomyopathy presenting with heart failure secondary to left ventricular systolic dysfunction toward the end of pregnancy or in the months following delivery, where no other cause of heart failure is found. It is a diagnosis of exclusion. The left ventricle may not be dilated but the ejection fraction is nearly always reduced below 45%” [7]. This statement leans toward PPCM as a diagnosis of exclusion and highlights the variety of presentations and unpredictability that is at the core of the uncertainty of PPCM. When compared to other heart failure causes, the main differentiator for a diagnosis of PPCM is the temporal relationship to the puerperal period. 

Common presentations of PPCM are progressive dyspnoea, chest pain, orthopnoea, persistent cough, abdominal pain, palpitations, abdominal discomfort, peripheral oedema. The majority of cases present in the postpartum period (78%), so much so that previously PPCM was known as postpartum cardiomyopathy [7,8]. 

### 1.2. Takotsubo Syndrome

Takotsubo syndrome (TTS) is a mostly reversible cause of left ventricular pump failure that began to be characterised in the 1990s by the Japanese physician Hikaru Sato. Japanese researchers discovered an increased incidence of patients with reversible cardiac failure following an earthquake in Japan [9,10]. The name “takotsubo” was given due to the appearances of the left ventricle in imaging bearing a resemblance to a round-bottomed pot with a narrow neck used by Japanese sailors to entrap octopuses [11,12]. As the recognition of TTS has grown, there have been different names used to describe the condition, such as ’transient left ventricular apical ballooning syndrome”, ”broken-heart syndrome” or “stress cardiomyopathy” in reference to the common presentation of TTS following a significant emotional or physical stressor [13]. 

Much like PPCM, the diagnostic criteria have undergone a multitude of revisions and subject to ongoing discussion and debate. Tracking the evolution of the diagnostic criteria will give us an insight into the changes in the thinking concerning TTS. The first widely accepted criteria, published by the Mayo Clinic, is summarised below [13]:

1. “Transient hypokinesis, akinesis, or dyskinesis of the left ventricular mid-segments with or without apical involvement; the regional wall motion abnormalities extend beyond a single epicardial coronary distribution; a stressful trigger is often, but not always present.
2. Absence of obstructive coronary disease or angiographic evidence of acute plaque rupture.
3. New electrocardiographic abnormalities (either ST-segment elevation and/or T-wave inversion) or modest elevation in cardiac troponin.
4. Absence of:
  - Phaeochromocytoma
  - Myocarditis”

Over time, thinking changed, and the takotsubo syndrome diagnosis has become more inclusive as the viewpoint has shifted toward takotsubo syndrome as a type of stress cardiomyopathy. Subsequent criteria have become far more inclusive of echocardiograph presentations that do not follow the classic apical ballooning picture. The most recently accepted and internationally quoted is the InterTAK diagnostic criteria [12], consisting of eight points:

1. “Patients show transient left ventricular dysfunction (hypokinesia, akinesia, or dyskinesia) presenting as apical ballooning or midventricular, basal, or focal wall motion abnormalities. Right ventricular involvement can be present. Besides these regional wall motion patterns, transitions between all types can exist. The regional wall motion abnormality usually extends beyond a single epicardial vascular distribution; however, rare cases can exist where the regional wall motion abnormality is present in the subtended myocardial territory of a single coronary artery (focal TTS).
2. An emotional, physical, or combined trigger can precede the takotsubo syndrome event, but this is not obligatory.
3. Neurologic disorders (e.g., subarachnoid haemorrhage, stroke/transient ischaemic attack, or seizures) as well as pheochromocytoma may serve as triggers for takotsubo syndrome.
4. New ECG abnormalities are present (ST-segment elevation, ST-segment depression, T-wave inversion, and QTc prolongation); however, rare cases exist without any ECG changes.
5. Levels of cardiac biomarkers (troponin and creatine kinase) are moderately elevated in most cases; significant elevation of brain natriuretic peptide is common.
6. Significant coronary artery disease is not a contradiction in takotsubo syndrome.
7. Patients have no evidence of infectious myocarditis.
8. Postmenopausal women are predominantly affected.” [14]

The InterTAK diagnostic criteria embrace takotsubo syndrome as a disease process that can have triggers and seeks to include patients that have preceding conditions that can serve as a trigger, such as phaeochromocytoma. The criteria also acknowledge the predominance of TTS in postmenopausal women.

“The International Expert Consensus Document on Takotsubo Syndrome” also concludes that 90% of patients experiencing TTS are female [14]. A comparison of the gender predominance in international registries revealed that women make up between 77% and 90% of patients in international registries. Multiple studies have demonstrated that 89% of the cohort in the large multi-centre trials are women [10,15]. This fact should not be ignored in the discussion concerning the aetiology of takotsubo syndrome, with some having theorised the declining oestrogen levels in postmenopausal women is a cause of its predominance in women. As oestrogen has been demonstrated to have a cardioprotective effect, the decline in oestrogen in a postmenopausal woman could leave her more vulnerable to myocardial stress. This, however, has been contested by a study that shows a significantly higher oestrogen (E2) level in patients experiencing takotsubo [16]. It is clear more scientific studies are required to determine the role oestrogen has in TTS, if any.

As seen with PPCM, TTS is a diagnosis of exclusion, and the diagnostic criteria are wide, and the main focal point is centred around echocardiography findings detecting the presence of left ventricular dysfunction. Interestingly, in the perinatal woman echocardiography findings, both are similar; so similar, in fact, that in a study comparing the echocardiograph findings there was a significant disagreement between cardiologists as to whether the appearance of the echocardiograph was attributable to typical features of PPCM or TTS [17]. TTS seems to exist on a spectrum of severity like most syndromes, with the severe end of the spectrum associated with higher morbidity or mortality and the other end with mild presentations that resolve quickly with minimal morbidity. 

Table 1 surmises the differences in diagnosis and presentation of PPCM and TTS. Note that the investigations findings, such as ECG findings and MRI findings, are highly dependent on which stage of the disease process the investigation is performed. It has been noted in several reviews that women present later with peripartum cardiomyopathy as the symptoms in their mildest form can often mimic late pregnancy symptoms. Therefore, on the whole, it would be reasonable to note that PPCM will often present later in the disease process than the hyperacute presentations that are more common in TTS. 

## 2. Materials and Methods

Compared to PPCM, TTS is a relatively new discovery. As expected, there has been much discussion concerning how the characteristics of TTS differ from all other heart failure diagnoses. The bulk of the discussion concerning establishing TTS as a distinct disease has been centred on distinguishing TTS from heart failure, secondary to acute myocardial infarction [40]. To such an extent that despite the changes over the years in the diagnostic criteria for TTS, the consistent cornerstone of diagnosing TTS has always included ruling out myocardial infarction by cardiac catheterization [14].

Some researchers have been concerned with the possibility of TTS occurring in the peripartum period being mistaken for PPCM and have coined such instances “peripartum takotsubo syndrome” (PTTS). This is where this literature review focuses, with the aim being to look at studies comparing cohorts of PPCM and TTS or describing takotsubo in the peripartum context and the ways TTS differs from PPCM. As established, the predominant population groups in which TTS occurs is postmenopausal women. This introduces a great deal of bias in the analysis of outcomes as the physiology of a pregnant/postpartum woman is not the same as a postmenopausal woman; therefore, the two groups are not equivalents. We sought to eliminate that issue by focussing on the pregnant/postnatal women as a population group. Allowing for a singular population group with similar physiology as the aim of the review is to contrast TTS and PPCM in the same population group. The pregnant/postnatal patients seemed ideal for this comparison as it is a population in which both TTS and PPCM can reasonably occur and, therefore, it would be possible to perform a like-by-like comparison. The methodology for this review strategy has been adapted from PRISMA and AMSTAR guidance [41].

The central question of the literature review is this: Are there any observed differences between patients diagnosed with peripartum cardiomyopathy compared with peripartum takotsubo syndrome?

### 2.1. Inclusion/Exclusion Criteria

To improve the reliability of the data set, we aimed to avoid case reports and included cohort sizes greater than five cases. Ideally, RCT and multicentre cohort studies would provide the most robust data set; however, TTS is rare, and TTS diagnosed in the peripartum patient is even less common. The criteria for the selection of studies were agreed upon by two reviewers, although the elimination of studies was performed by a single reviewer.

Cohort studies (whether retrospective or prospective) included must have clear definitions of PPCM and TTS. Furthermore, they should have a consistent, robust echocardiogram criterion for determining if a study participant has TTS or PPCM. There must also be a strategy for ruling out other causes of heart failure in the perinatal patient. All studies included within the review must have patients diagnosed with PPCM and patients diagnosed with peripartum TTS within their patient cohort. Therefore, a comparison in outcomes, clinical characteristics, and diagnostic markers can be performed. An established consistent protocol with the same reviewers for all patients must be present to reduce the selection bias; for example, diagnostic imaging should be reviewed by two cardiac imaging specialists for all patients to determine whether the patient will be allocated to the PPCM or TTS group.

The studies must be published in a peer reviewed journal, and the original publication language must be in English.

### 2.2. Search Strategy

**Figure 1:**
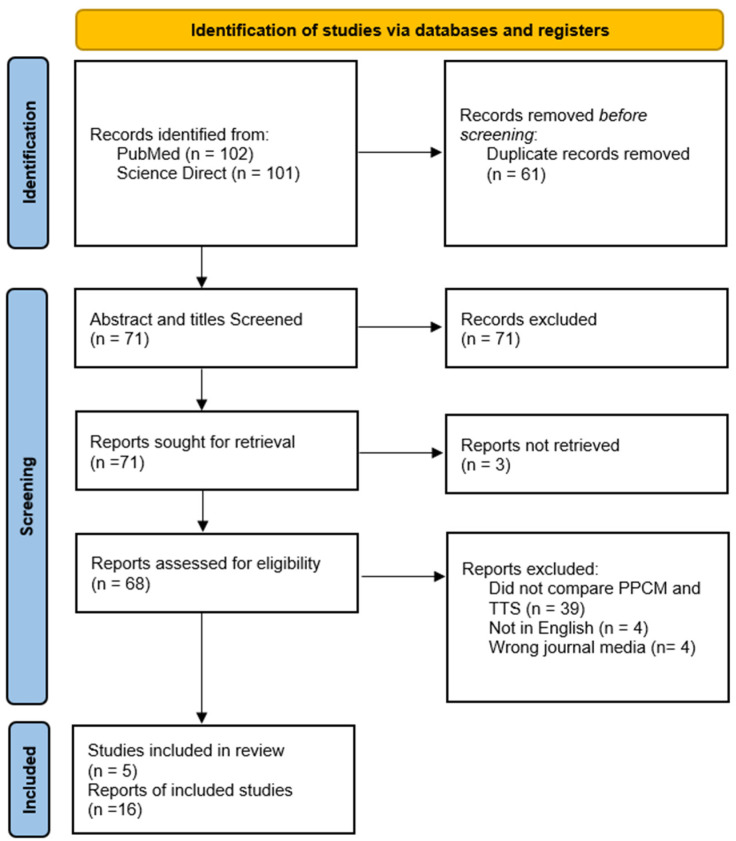
PRISMA Diagram search strategy.

The PRISMA guidance dictated the search methodology. Search keywords used were “Peripartum cardiomyopathy” AND “Takotsubo syndrome” OR “Peripartum takotsubo syndrome”. [Figure 1] demonstrates the literature search according to PRISMA guidelines [42]. A single reviewer was used for the whole process as is acceptable for rapid reviews.

## 3. Results

Table 2 summarises the key findings from both cohort studies included in the review.

Both studies demonstrate the difficulty in distinguishing PPCM from TTS in the perinatal patient. As both TTS and PPCM are diagnoses of exclusion, the methodology for determining which cohort a patient belongs to in both studies seemed largely focused on the echocardiograph features: typical (apical ballooning) or atypical (reverse/inverted ballooning) indicates TTS and PPCM seem to encompass any other echocardiograph pattern with a reduced ejection below 45%.

Both studies have a relatively small cohort size, which is expected, given the rarity of PPCM and TTS in the peripartum patient. Furthermore, all takotsubo patients are postnatal in the Yang et al. study. This difference may be as some have suggested; i.e., that TTS is mainly found in postpartum patients due to the sudden fall in oestrogen following the expulsion of the placenta after birth [44]. There are many difficulties with this line of thinking. Firstly, there is conflicting evidence concerning the role of oestrogen in TTS, and secondly, there are clear reported cases of TTS in the postpartum and antepartum patient, so a largely postnatal TTS patient population would skew the data and the outcomes [44].

In the Kim et al. study, patients classified as TTS in the peripartum period had a statistically significant difference in the number of near-death events and yet a 100% recovery rate with an ejection fraction above 55% over 1 month following the diagnosis. This presents a conundrum, as that does not match the non-pregnant population in which recovery within 1 month has much lower rates [34,38].

## 4. Discussion

The predominant method of distinguishing TTS from PPCM in both studies is “echo findings”. This is not unique to these studies; in fact, many of the case studies on peripartum takotsubo distinguished TTS from PPCM by the echocardiograph features alone. However, as we have discussed, the InterTAK diagnostic criteria accepts there are many other echocardiograph presentations of TTS apart from the typical left ventricular regional wall motion abnormalities, such as apical ballooning pattern [12]. This means that an echocardiograph or cardiac MRI pattern that would be considered typical of PPCM, such as left ventricular regional wall abnormalities, could be considered as TTS by the InterTAK criteria or PPCM by the ESC criteria. This leaves room for diagnostic ambiguity as evident by the disagreement whether specific patients would be considered PPCM or TTS by the cardiologists reviewing the echocardiographs in the Kim et al. study. Both disease entities carry the same long-term risks based on the extent of fibrosis based on late-gadolinium enhancement assessments [22,31].

Both studies adhere to the view that all PPCM patients must have an ejection fraction below 45%. The position statement from ESC is clear in stating there are cases where the ejection fraction is not below 45%. However, in multiple PPCM studies, patients with an ejection fraction above 45% are excluded. For example, in Peripartum Cardiomyopathy in Nigeria (PEACE), one of the largest single-country cross centre cohort studies, patients with an ejection fraction greater than 45% were excluded from the study completely, which accounted for just over 14% of patients recruited to the study who were given a diagnosis of PPCM at their local centre [45]. This pattern is repeated in many trials and even in international cross centre studies such as ESC EURObservational Research Programme PPCM registry, which used LVEF of less than or equal to 45% [46].

This creates a confirmation bias of sorts in the literature concerning PPCM. Perhaps in cases where the ejection fraction is mildly reduced yet above 45% with symptomatic patients, it is likely to be attributed by the clinician to TTS disease process rather than PPCM or an alternate cause of reversible cardiac failure. Additionally, due to the relative rarity of sudden, onset heart failure occurring in the peripartum period, much of the literature is case reports, meaning the reported cases are cases with a severely reduced ejection fraction. Conversely, as the definition for TTS becomes wider, there is not an accepted threshold for ejection fraction that would represent TTS. This leaves room for a greater variety in the patients and, therefore, will affect all the observed outcomes. This may be a contributor in the better reported outcome of TTS patients in the Kim et al. study.

### Is This All One Syndrome?

As shown in Table 1, and discussed above, objective diagnostic features around TTS and PPCM can be similar, including ECG patterns, Echo changes, clinical biomarkers, and cardiac MRI findings.

Applying a diagnosis retrospectively (Table 2) based on the recovery of left ventricular function is not helpful nor useful to clinicians when managing a sick and worried patient contemporaneously. Furthermore, the recovery of left ventricular function relates more to the severity of the underlying disease presentation than the semantic application of PPCM or peripartum Takotsubo labels.

A fundamental issue in terms of the behavioural aspects of differentiation between PPCM and TTS is how does the ambiguity affect clinical decision-making? It is clear from the evidence reviewed that there is, at least, considerable overlap between these two syndromes. It may also be gleaned from the evidence presented that these conditions represent one syndrome that is expressed in two distinct populations: those that are perinatal and those that are not perinatal. The implications of this are that one group will be assessed and managed by a team led by obstetricians, while the other is managed by cardiologists. However, a compelling rationale for clarity over the substance of a discrete underlying unitary condition is that recent innovations in structural equation modelling (SEM) have been able to establish whether distinct medical conditions may have the same underlying aetiology. This is of fundamental importance when the treatment interventions are different between groups. As an example, chronic fatigue syndrome/myalgic encephalomyelitis (CFS/ME) was, for many years, believed to be a condition largely of psychiatric origin. However, studies which have examined the symptom profile of ME/CFS in comparison with illness circumscribed by autoimmune aetiology (for example, fibromyalgia) have indicated that these may be the same condition [47]. Therefore, it is in this instance that the belief about the aetiology, psychiatric vs. autoimmune arose, which guided fundamentally different treatments for these two conditions for many years, and to some degree, still do. The same approach used by McKay et al. [48] can also be used to evaluate the symptom profiles between patients with PPCM and those with TTS to determine, with a significant degree of confidence, whether this is indeed one and the same underlying pathology with a common aetiology. Consequently, statistical methods such as SEM developed within the behavioural sciences may be of huge benefit in understanding the common aetiological heritage that may underpin both of these conditions. Using these approaches (SEM), we can thus generate a robust and plausible evidence base that contributes to enhancing patient care and improving clinical outcomes.

## 5. Conclusions

There are many areas in which there is significant overlap between PPCM and TTS. One could even hazard to say there are more similarities between PPCM and TTS than differences. Further research is required to definitively unify PPCM and TTS or reduce the diagnostic ambiguity.

Supportive care is the mainstay management in patients with both PPCM and TTS: this would be comprised of the typical heart failure regime. In PPCM, bromocriptine has proven highly beneficial in improving outcomes. This has been based on the role of prolactin in pathogenesis of PPCM [23]. However, there is no current evidence base exploring the use of bromocriptine in TTS in the peripartum patient. Equally, the role of oestrogen in PPCM remains to be explored. Perhaps considering the two conditions to be along the same spectrum, much like what was done with CFS and ME, may be beneficial to aide research and innovation in demystifying the pathogenesis and improving the understanding to counsel women appropriately and provide better care, especially as there is little evidence determining the risk of reoccurrence of TTS in the pregnant population.

## Figures and Tables

**Table 1:** PPCM and TTS comparison table.

	Peripartum Cardiomyopathy (PPCM)	Takotsubo Syndrome (TTS)
**Demographics**	Pregnant women in the last trimester up to five months postpartum [8]	>89% of cases occur in women. Usually, postmenopausal [15,18]
**Symptoms**	Shortness of breath on exertion, chest pain, paroxysmal nocturnal dyspnoea, orthopnoea collapse and terminal arrhythmia [19,20,21]	Shortness of breath on exertion, chest pain, paroxysmal nocturnal dyspnoea, orthopnoea, collapse and terminal arrhythmia [22]
**Presentation**	Can present acutely however late presentation and delayed diagnoses are common due to symptoms can be attributed to postpartum or late pregnancy symptoms [19,21,23]	Tend to present acutely, usually sudden onset Can occur in patients in the context of acute illness such as sepsis [22,24]
**Echocardiograph features**	Temporal dependant Global hypokinesia Left ventricular and right ventricular dilatation and/or dysfunction, functional mitral and/or tricuspid regurgitation, pulmonary hypertension, and left atrial or bi-atrial enlargement. Systolic dysfunction Intracardiac thrombus [19,25]	Temporal dependant Symmetrical regional abnormalities involving the midventricular segments of the anterior, inferior, and lateral walls Left ventricular dysfunction (hypokinesia, akinesia, or dyskinesia) presenting as apical ballooning or midventricular, basal, or focal wall motion abnormalities [14,26] Intracardiac thrombus [14,22]
**Electrocardiograph features**	Normal ECG, Sinus tachycardia Pathologic Q-waves, ST depression, T-wave abnormalities, 2nd- or 3rd-degree atrioventricular block, complete left or right bundle branch block, atrial fibrillation or flutter, and frequent atrial or ventricular ectopy [7,27]	Hyperacute: ST-segment elevation, ST-segment depression, and QTc prolongation Late features: T-wave inversion [12,22,28]
**Cardiac Magnetic Resonance Imaging features**	Acute presentation: High-signal T2 suggestive of oedema [25,29,30] Regional wall motion abnormalities [29] Late-Gadolinium enhancement sometimes seen—non-specific distribution. [29,31,32] Late Gadolinium enhancement confers worse recovery [31]	Acute: High-signal T2 suggestive of oedema [33,34], late Gadolinium enhancement suggestive of fibrosis is usually absent in the acute stage but can be present [12,22]. Late Gadolinium enhancement suggests more severe disease and less recovery. [22,34]
**Aetiology**	Prolactin mediated [19] Inflammation Two hit mechanism, genetic pre-disposition, and precipitating event Mostly unknown	Neuroendocrine storm (adrenaline, noradrenaline) [24] inflammation Reduced oestrogen levels Mostly unknown
**Average Time for recovery**	Highly variable [35] LVEF recovery time: 34% in 6 months 47% in 1 year 71% in 5 years [20] Mortality rate 1.6% to 27.6% [7]	Mean LVEF recovery at 60 days [18] Partial recovery rate 16–30%, persistent reduced LVEF associated with multiple co-morbidities [34,36,37] Late (>10 days) recovery 53% [38] Early (<10 days) recovery 47% [38] Mortality rate 4.5–5.6% [18,39]
**Biochemical markers**	BNP, Troponin, CRP microRNA-146a, cathepsin D, and interferon-gamma [7]	BNP, Troponin, CRP [12]

**Table 2:** Summary of results.

	Yang, W-I et al. (2019) [43]	Kim, D-Y et al. (2020) [17]
Study design	Retrospective observational single centre	Retrospective observational single centre
Number of patients	37 21 (PPCM) 16 (TTS)	31 21 (PPCM) 10 (TTS)
PPCM definition	LVEF < 45%, 3rd trimester of pregnancy, 6 months postpartum, left ventricular global hypokinesia	LVEF < 45%, 3rd trimester of pregnancy, 6 months postpartum, left ventricular global hypokinesia
TTS definition	Regional wall abnormalities, LVEF < 45%	Transient regional wall motion abnormalities (RWMAs) that extended beyond a single epicardial vascular distribution during the last month of pregnancy or within 5 months after delivery, with either electrocardiographic abnormalities or modest cardiac troponin elevation
Similarities between cohorts	Clinical characteristics, Biochemical markers	No statistically significant difference in the mode of delivery Similar rise in biomarkers
Differences between cohorts	Greater parity in TTS Earlier onset of symptoms in TTS Higher LVEF with quicker recovery Complete resolution of EF for all TTS patients at 1 month	Greater near-miss death events in TTS

## Data Availability

Not applicable.
